# Visible light-driven photodynamic therapy for hypertrophic scars with MOF armored microneedles patch

**DOI:** 10.3389/fchem.2023.1128255

**Published:** 2023-02-16

**Authors:** Danyang Chen, Yixuan Zhang, Wei Long, Langjie Chai, Thazin Phoone Myint, Wei Zhou, Ling Zhou, Min Wang, Liang Guo

**Affiliations:** Department of Plastic Surgery, Zhongnan Hospital of Wuhan University, Wuhan, China

**Keywords:** Photodynamic therapy, Metal-organic frameworks, chloroquine, microneedles patch, photosensitizers

## Abstract

Photodynamic therapy (PDT) is widely used for the treatment of hypertrophic scars in clinical practice. However, the low transdermal delivery of photosensitizers in scar tissue and protective autophagy induced by Photodynamic therapy greatly reduces the therapeutic efficiency. Therefore, it is necessary to deal with these difficulties for overcoming obstacles in Photodynamic therapy treatment. In this study, a photosensitizer with photocatalytic performance was designed and synthesized using innovative MOFs (metal-organic frameworks). Additionally, the MOFs, together with an autophagy inhibitor chloroquine (CQ), was loaded in a high mechanical strength microneedle patch (MNP) for transdermal delivery. With these functionalized MNP, photosensitizers and chloroquine were delivered deep inside hypertrophic scars. Inhibition of autophagy increases the levels of reactive oxygen species (ROS) under high-intensity visible-light irradiation. Multiprong approaches have been used to remove obstacles in Photodynamic therapy and successfully enhance its anti-scarring effect. *In vitro* experiments indicated that the combined treatment increased the toxicity of hypertrophic scar fibroblasts (HSFs), downregulated the level of collagen type I expression as well as transforming growth factor-β1 (TGF-β1)expression, decreased the autophagy marker protein LC3II/I ratio, increased the expression of P62. *In vivo* experiments showed that the MNP had good puncture performance, and significant therapeutic effects were observed in the rabbit ear scar model. These results indicate that functionalized MNP has high potential clinical value.

## 1 Introduction

Hypertrophic scarring (HS) is the result of a transitional healing response to trauma, inflammation, and burns and is a common disease (Grabowski et al., 2020; Wang et al., 2020). Microscopically, scar tissue shows over-proliferation of fibroblasts, transitional transition deposition of collagen, proteoglycan, and fibronectin in the extracellular matrix (ECM), and arrangement of disordered collagen fibers (Tosa and Ogawa, 2020; Ogawa et al., 2021). Hypertrophic scars are extremely harmful, as they not only affect the patient’s appearance but also induce local tissue redness, thickening, pain, and itching, and even lead to joint dysfunction and skin cancer (Kessel and Reiners, 2020). The recurrence of hypertrophic scars after surgical treatment is one of the biggest challenges in scar treatment. Thus, non-surgical treatments including intra-scar injection of triamcinolone acetonide or 5-fluorouracil, compression therapy, laser therapy, radiotherapy, and photodynamic therapy (PDT) were advocated as combined therapies (Grabowski et al., 2020; Ogawa et al., 2021). Among them, PDT has been proven to be a safe therapy with mild hyperplasia, reduced pruritus and pain caused by scars, and increased scar softness (Ud-Din et al., 2013; Zhang et al., 2019; Tosa and Ogawa, 2020). However, the efficiency of PDT for hypertrophic scars is hindered by three main issues:1) poor transdermal delivery of photosensitizers in scar lesions (Tosa and Ogawa, 2020). 2) traditional photosensitizers, such as 5-ALA, are acquired for excitation light with special wavelengths, which are hardly accepted by patients because it takes too much time in the treatment process with an annoying light source (Chen D. et al., 2021). 3) Cytoprotective lysosomal autophagy occurs inside scar cells, resulting in PDT resistance (Martins et al., 2020). To solve these difficult issues, high photosensitizer transdermal efficiency, low levels of cell autophagy, and patient-acceptable light sources for PDT are required.

To improve the penetration efficiency of photosensitizers into scar tissue, many transdermal systems have been developed (Zhang et al., 2019; Chen et al., 2020; Yu et al., 2022). Among them, microneedles patch (MNP) comprising a transdermal patch and hypodermic needles can significantly improve the permeability of drugs (Indermun et al., 2014; Yin et al., 2022). It is also considered an effective method of transdermal drug delivery and a milestone in the treatment of skin diseases. However, few studies have focused on microneedles in scar treatment, mainly because of the difficulty in synthesizing biodegradable microneedles with a high density (Chen et al., 2022). In our previous study (Huang et al., 2022a), microneedles with an appropriate ratio of gelatin and starch were constructed for developing high-strength microneedles was investigated. And we will further discuss the microneedles’ potential transdermal photosensitizer delivery capability and its therapeutic effects.

Photosensitizers require a high light source and have certain limitations in terms of clinical treatment. The development of photosensitizers may provide a solution to this problem by modifying them to achieve PDT under visible light (Zheng et al., 2021; Fan et al., 2022; Liu et al., 2022). In contrast to many other PDT for deep-seated tumors, PDT for skin lesions do not restrict to limited well-penetrated near-infrared light. Studies reported that yellow LED-light, blue of argon lasers and other invisible light sources were chosen for superficial lesions, not confined to abnormal scar in PDT, and resulting in good therapeutic effects (Hu et al., 2017; Tosa and Ogawa, 2020). Furthermore, because of less painful, more convenient, daylight photodynamic therapy (dPDT) by using sunlight as a light source became an effective alternative therapy for many superficial skin lesions (Lee C. N et al., 2020). To realize the scar PDT by visible sunlight, the photosensitizers must be well designed with an ability of generating high level of hydroxyl radicals under visible light irradiation. Inspired by our collaborators’ work (Jiang et al., 2020), we choose well designed MOFs (Metal-organic framework) as photosensitizer. Metal-organic frameworks (MOFs), also known as coordination polymers, are formed by strong coordination bonds between organic ligands and metal nodes. They can be designed and synthesized based on the demand of the application (Yang and Yang, 2020; Zheng et al., 2021). In particular, they have a significant potential in the field of photocatalysis and biomedical (Jiang et al., 2020). Building upon the work of collaborators who synthesized MIL-101-Cr (Jiang et al., 2020), MOFs can be functionalized as photosensitizers with good photocatalytic properties when exposed to visible light. These MOFs have the potential to be used in photodynamic therapy (PDT) for scar treatment.

Autophagy is a catabolic mechanism and it is very important for keeping the balance of the intracellular environment, removing damaged organelles, and degrading cytotoxic molecules (Lee S. et al., 2020). By lysosome-dependent mechanism, autophago-lysosomes degrade damaged organelles and proteins. Thereby the dynamic balance and integrity of cells was maintained (Klionsky and Emr, 2000). PDT-induced cell damage activates a variety of protective cell autophagy pathways, resulting in PDT resistance and a reduction in PDT efficacy (Liu et al., 2019; Martins et al., 2020). Ouyang et al. found that during the treatment of keloids with PDT, the SIRT1-SIRT3-SOD2-mROS-dependent autophagy pathway was downregulated, resulting in a reduction in the level of mitochondria-derived reactive oxygen species (ROS), which in turn reduced PDT-induced cell death (Liu et al., 2019). Therefore, PDT may effective enhanced when combined with autophagy inhibitor, which effectively inhibited autophagy by destroying the structure and function of lysosomes, leading to aggregation of autophagy-lysosome.

**SCHEME 1:**
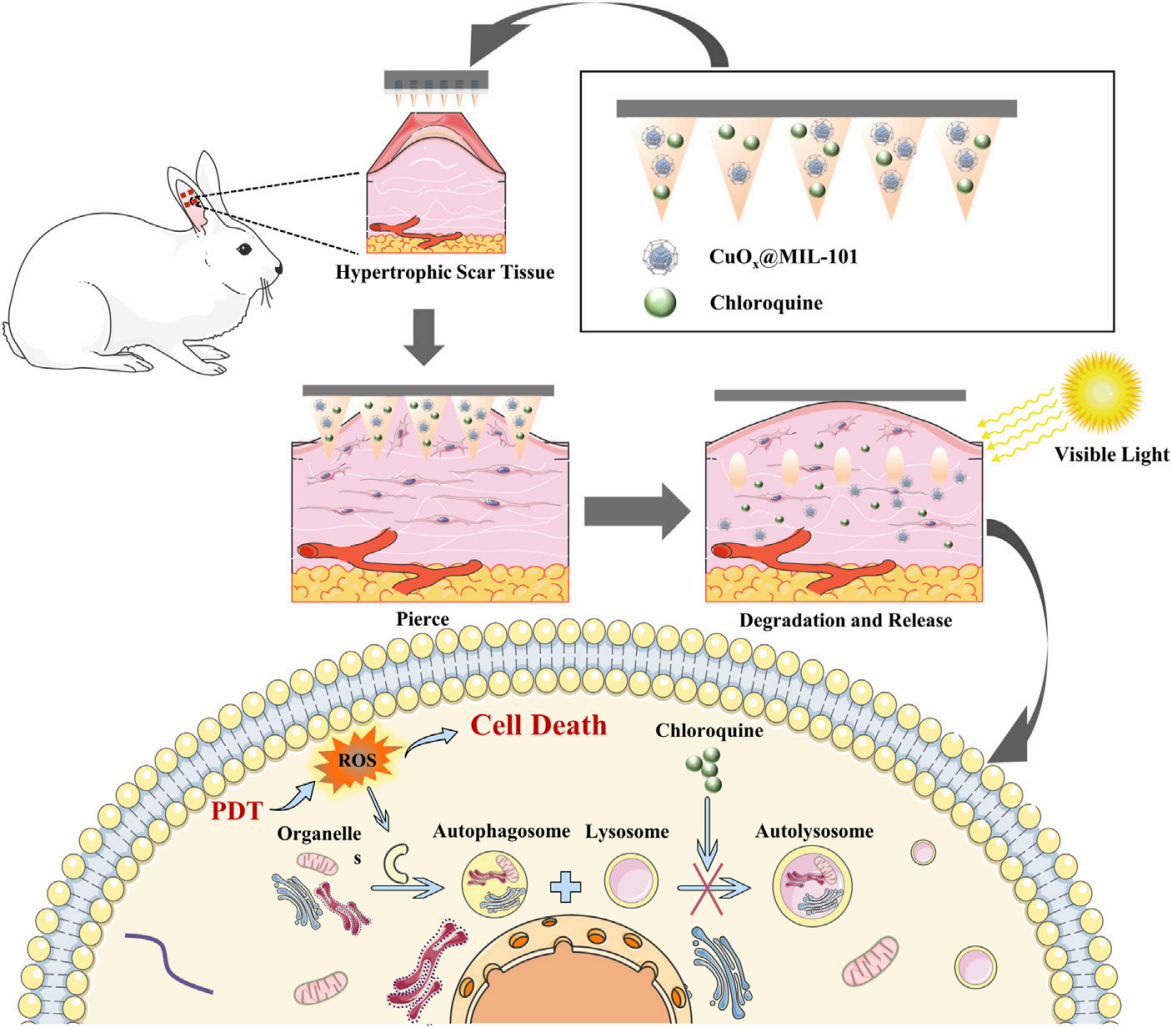
Illustration of synthesis of CuO_X_@MIL-101 and functional MNP mechanism of PDT for the hypertrophic scar.

In our study, we designed an innovative MNP with good biocompatibility loaded with MOFs and autophagy inhibitors that could effectively overcome the skin stratum corneum barrier and deliver photosensitizers to the deep lesions of HS. The photosensitizer CuO_x_@MIL-101 could efficiently generate ROS in PDT with visible light, which is easy to acquire and acceptable for patients. Furthermore, the enhanced PDT therapeutic efficiency of an autophagy inhibitor and its mechanism of action were investigated. To summarize, these novel functionalized MNP with efficient transdermal delivery and small invasion MNP will offer a prospective strategy for PDT in hypertrophic scars (Scheme 1).

## 2 Materials and methods

### 2.1 Materials

All the chemicals were used without further purification. Terephthalic acid (BDC), N, DMF (99.8%) (HF, 37wt%), n-hexane (CH3(CH2)4CH3, 98.5%), and ethanol (C_2_H_5_OH, 99.7wt%) were purchased from Sinopharm Chemical Reagent Co. Ltd. (Shanghai, China). Chromic nitrate non-ahydrate (Cr(NO_3_)_3_ ·9H_2_O), copper chloride (CuCl_2_), and sodium hydroxide (NaOH) were purchased from Aladdin (Shanghai, China). Gelatin and starch were purchased from Sigma–Aldrich (St. Louis, MO, United States of America). Rhodamine 6G was purchased from Aladdin (Shanghai, China). Chloroquine was purchased from Shanghai Macklin Biochemical Co. (Shanghai, China), A 300w xenon lamp (Beijing Perfect Light, Beijing, China) was used as the light source.

### 2.2 Synthesis and characterization of CuO_x_@MIL-101

#### 2.1.1 Synthesis of MIL-101

MIL-101(Cr) was synthesized using a modified hydrothermal method reported by Férey (Férey et al., 2005). Typically, 332 mg of terephthalic acid (BDC) and 800 mg of Cr(NO_3_)_3_·9H_2_O were suspended in 14 ml of H_2_O, followed by the addition of HF (0.1 ml) (37%). The mixture was heated at 220°C for 8 h in a high-pressure autoclave and then cooled to 25°C to obtain a green powder. The product was washed with DMF and ethanol at 80°C for 30 min. This procedure was repeated at least three times to remove unreacted terephthalic acid and DMF. Subsequently, the green powder was activated at 150 °C for 12 h under a vacuum for further use.

#### 2.1.2 Synthesis of CuO_x_@MIL-101

Activated MIL-101 (100 mg) was suspended in 40 ml of dry n-hexane, a hydrophobic solvent, and the mixture was sonicated for 2 min. Then 0.1 ml CuCl_2_ solution (0.25 M) and 0.1 ml NaOH solution (0.5 M) were added dropwise into the mixture at a rate of 5 μl/min under continuous stirring. N-hexane was carefully removed by centrifugation, and the green powder was dried at room temperature. Finally, the products were obtained by heating the green powder at 100°C for 12 h.

#### 2.1.3 Characterization test of MIL-101 and CuO_x_@MIL-101

The morphologies of MIL-101 and CuO_x_@MIL-101 were observed using scanning electron microscopy (SEM, FEI Verios-460), and elemental mapping of a bright-field scanning SEM (OXFORD Ultim Extreme, X-Max 80, Abingdon, United Kingdom) was used to confirm the composition of CuO_x_@MIL-101. The crystallinity of the MOFs was determined using X-ray diffraction (XRD) (Rigaku SmartLab diffractometer with filtered Cu Kα radiation [λ = 1.5405 Å]). Moreover, the pore sizes of MIL-101 and CuO_x_@MIL-101 were determined using N_2_ adsorption experiments (Ipore 400 instrument [PhysiChem Instruments Ltd. Beijing, China]). Additionally, the ROS production of CuO_x_@MIL-101was measured. Subsequently, CuO_x_@MIL-101 was mixed with an aqueous solution of terephthalic acid (PTA) and reacted at room temperature in the dark. 300 W Xe lamp (Beijing Perfect Light) and light filter (λ > 420 nm) were irradiated at the top of the solution. The photoruminescence (PL) spectra of the solution was detected every 15 min by the fluorescence spectrophotometer (Hitachi F-4600 Model F-4600 FL Spectrophotometer) under excitation with UV light at 315 nm.

### 2.3 Cellular studies

#### 2.3.1 Primary culture of hypertrophic scar fibroblasts

This study was approved by the Medical Ethics Committee, Zhongnan Hospital of Wuhan University (Ethics No. 2021115). Scar tissue specimens were obtained from five surgical patients, and informed consent was confirmed by the patients for experimental use. Cells from passages three to six were used for subsequent experiments.

#### 2.3.2 Evaluation of biocompatibility of CuO_x_@MIL-101 *in vitro*

To access the migration of HSFs treated with different concentrations of CuO_x_@MIL-101 (1, 10, 100,1000 μg/ml), the scratch-wound assay was perfomed. The Cell Counting Kit 8 (CCK-8) assay (CK04; Dojindo, Kumamoto, Japan) was used to assess the viability of HSFs after treatment with these concentrations of CuO_x_@MIL-101 for 24 h, 48 h, and 72 h. A hemolysis test was also performed to evaluate the safety of CuO_x_@MIL-101 before its use *in vivo*. Detailed experimental methods were in the Supplementary Methods. This study was performed with the approval of the Experimental Animal Welfare Ethic Committee, Zhongnan Hospital of Wuhan University, Wuhan University (Approval No. ZN2022123).

#### 2.3.3 *In vitro* anti-scarring efficacy

Cytotoxicity assay was performed using the Cell Counting Kit 8 (CCK-8) assay (CK04; Dojindo, Kumamoto, Japan). Briefly, HSFs were seeded in 96-well culture plates (5 × 10^3^ cells/well) and allowed to incubate for 24 h. Cells were treated according to grouping (A:3 μg/ml MOFs +10 μg/ml CQ; B:3 μg/ml MOFs; C:10 μg/ml CQ; D: blank control group) and cultured for 12 h, then groups A and B were irradiated using a Xe lamp (Beijing Perfect Light) (30–50 mW/cm^2^) for 5 min. The cells were incubated for 24 h. A CCK-8 assay was performed. Cell proliferation was determined by measuring the absorbance of the different treatment groups using a microplate reader (Thermo Fisher Scientific, Waltham, MA, United States of America).

#### 2.3.4 Intracellular ROS generation

Intracellular ROS was detected by oxidation sensitive dihydroethidium (DHE, Beyotime Institute of Biotechnology, Jiangsu) fluorescent probe. The HSFs were cultured in six-well plates and treat with MOFs + Light and MOFs + CQ + Light. Following treatment, cells were harvested and co-cultured with 10 mM DHE at 37°C for 20 min according to instructions. With microplate reader, the intracellular ROS was measured. Fluorescence microscope (Zeiss, Jena, Germany) and flow cytometer (Beckman Coulter, Inc.) were used to observe intracellular ROS levels.

#### 2.3.5 RT-PCR assay

The mRNA expression of TGF-β1 and Collagen I in fibroblasts was detected using RT-PCR. Fibroblasts were cultured according to the grouping conditions described in section 2.3.3. Cells were washed twice with PBS, total RNA was extracted using Trizol reagent, and the RNA concentration was measured by spectrophotometry.

#### 2.3.6 Western blot analysis

The western blotting assay was carried out to detect expression levels of autophagy-related proteins. The total protein of cells in the different treatment groups was extracted, separated using dodecylsulfo-polyacrylamide gel electrophoresis, transferred to polyvinylidene fluoride membranes, and mixed with anti-LC3I, LC3II, and P62 at 25°C for 1 h. The gray value of total protein was normalized to that of GAPDH, and the western blot bands were densitometrically analyzed using ImageJ software.

### 2.4 Synthesis and physicochemical properties of microneedle patch

#### 2.4.1 Fabrication and characterization of MNP

Microneedle patch (MNP) was prepared using a two-step pouring method as our previous study (Huang et al., 2022a). Gelatin and starch were mixed in a ratio of 2:1 and heated at 90°C. Different drugs (group A, 800 μg/ml MOFs; group B, 800 μg/ml MOFs +1.5 μg/ml CQ) were added to the mother liquor. The mixture was evenly poured into a mold. Each pour was centrifuged to remove bubbles (3000–10000 rpm) and then concentrated at 28°C–35°C for 2–3 h. This process was repeated thrice until the mold was filled with a gel. Then, the mixture without MOFs and CQ was pipetted on the surface of the MNP mold, and the substrate was prepared by centrifugation (3000–10000 rpm) in the same protocol (Figure 3A). The molded MNP was carefully removed after drying overnight for 12 h. A scanning electron microscope (SEM) was used to detect the morphology of the MNP. Young’s modulus was measured to test the mechanical strength of the MNP. To further elucidate mechanical strength and transdermal delivery capability of MNP, MNP were punctured on rabbit ear scars for 10 min. They were then fixed in 4% paraformaldehyde for 18 h to further evaluate the mechanical properties of MNP. Samples were embedded in oct compound (SAKURA Tissue-Tek^®^, Torrance, CA, United States of America) and cut into 10 μm sections perpendicular to the skin surface using a microtome (Leica RM 2245, Wetzlar, Germany).

### 2.5 Evaluation of *in vivo* efficacy of rabbit ear scar model

#### 2.5.1 Establishment of rabbit ear scar model

To further evaluate the efficacy of photodynamic therapy combined with microneedle therapy on scars, we established a rabbit ear scar model. All procedures for experimental animals were approved by the Experimental Animal Welfare Ethic Committee, Zhongnan Hospital of Wuhan University (Approval No. ZN2022123).

12 Japanese white rabbits weighing 2500–3000 g were chosen for this study. Sodium pentobarbital (1%, 3 ml/kg) was injected into the ear vein for anesthesia. Four squares of 1 × 1 cm size, 4 cm from the ear tip, symmetrically distributed in pairs were marked. The area was pressed to stop bleeding and wrapped with gauze. Dressings were changed every 2 days after modeling, scabs were gently removed on day 7, and modeling was completed on day 21.

#### 2.5.2 Therapeutic effect of MNP on rabbit ear scar

The treatment efficiency for hypertrophic scar by of MNP was explored in the rabbit HS model. HSs were randomly divided into five different treatment groups. The blank control group was covered with gauze. In the MNP treatment group, the patch was pressed with the thumb and fixed with adhesive tape to immobilize (Supplementary Figure S1). Each group contained five HSs. All groups were given corresponding treatments (blank control, MOFs MNP, MOFs + light, MOFs MNP + Light, and MOFs MNP + CQ + Light) on the 1st, 8th, and 15th day after modeling. HS photographs were taken before and 1 week after each administration. Photographs were taken using a camera phone (Mi 10 Pro) to access the rabbit ear scar model during the treatment.

#### 2.5.3 Histological analysis and immunostaining

At the end of the treatment, the rabbits were sacrificed and ear scar specimens were collected and fixed with 4% (v/v) paraformaldehyde. All specimens were stained with H&E and Masson’s trichrome. Epithelialized collagen regeneration in the sample tissue was observed using inverted and upright fluorescence microscopy (Olympus BX51).

The scar hyperplasia index (SEI) value was calculated to quantify scar formation. The SEI was calculated as the mean of the five HSs in each group. Scarring was assessed as having an SEI value >1.5. Collagen deposition levels in HSs were assessed using collagen volume fraction (CVF). CVF (the ratio of collagen area to the total area in Masson-stained images) was calculated using ImageJ software, and five HSs were used for CVF analysis in each treatment group.

### 2.6 Statistical analysis

All data were statistically analyzed using one-way ANOVA, and t-tests. GraphPad Prism v. 9.4 (GraphPad Software, La Jolla, CA, United States of America) was used to analyze data and draw graphs, and all data are presented as mean ± standard error of the mean. This study used at least three independent samples for analysis, and the results were considered statistically significant at *p* < 0.05.

## 3 Results and discussion

### 3.1 CuO_x_@MIL-101 fabrication and characterization

**FIGURE 1:**
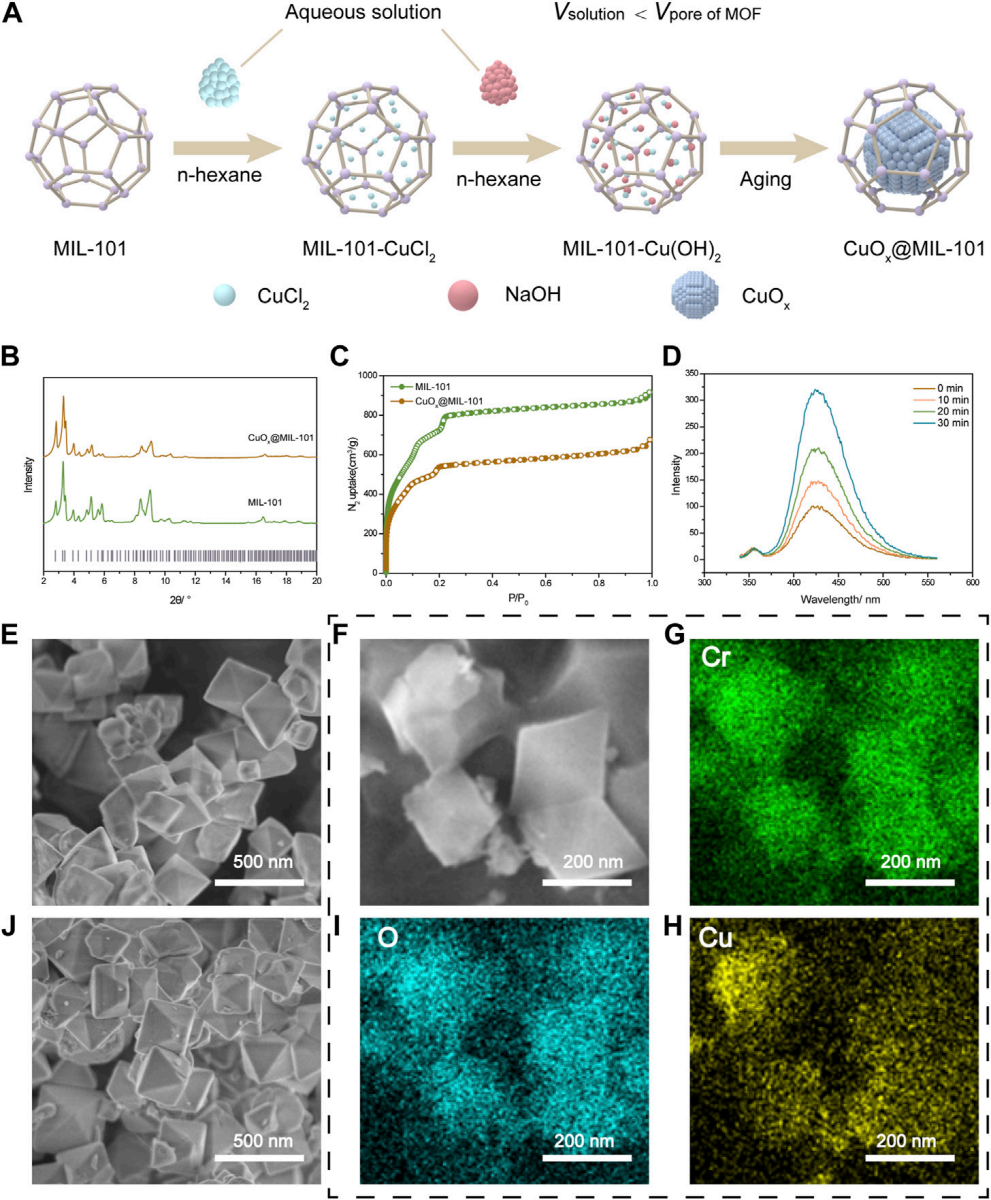
CuO_X_@MIL-101 nanoparticles characterization and its property of generating free radicals. **(A)** Schematic of the production of CuO_X_@MIL-101. **(B)** XRD X-ray diffraction (XRD) patterns of simulated MIL-101 and CuO_x_@MIL-101 **(C)** N2 isotherms of MIL-101 and CuO_x_@MIL-101. **(D)** ROS production was measured by the fluorescence spectrophotometer. **(E, J)** SEM images of MIL-101 and CuO_X_@MIL-101 (scale bar = 500 nm). **(F–H)** Elemental mapping of a bright-field scanning SEM images of the distributions of chromium (Cr), copper (Cu), and oxygen **(O)** within the MIL-101 crystals (scale bar = 200 nm).

We prepared supported CuO nanoparticles by the double-solvent method (Aijaz et al., 2012), as depicted in Figure 1A. The windows size of mesopore over MIL-101 was 1.2 nm and 1.6 nm. Both mesopore window sizes were larger than the diameters of Cu2+ and OH-, indicating the possibility of producing Cu(OH)_2_ within the pores of the MOFs backbones (Férey et al., 2005). The PXRD patterns of the pure MIL-101 and CuO_x_@MIL-101 are demonstrated in Figure 1B. All the diffraction peaks, as well as the Full-Width Half-Maximum (FMHM) values of the CuO_x_@MIL-101 composites, were similar to those of pristine MIL-101, which demonstrated that this loading process did not damage the crystallinity of the MOFs, as can be seen from the SEM images of MIL-101 (Figure 1E) and CuO_x_@MIL-101 (Figure 1J). Figure 1C shows N_2_ isotherms of these two MOFs. In comparison with the adsorption platform and BET surface area, there was a considerable decline in the platform from 800 cm^3^/g to 500 cm^3^/g and the surface area from 2790 m^2^/g to 2150 m^2^/g after impregnation with CuO. The composition of CuO_x_@MIL-101 was confirmed by elemental mapping using bright-field scanning SEM **(** Figure 1F–I), which show homogeneous distributions of chromium (Cr), copper (Cu), and oxygen O) within the MIL-101 crystals, further validating the successful generation of these CuO nanoparticles in the presence of MIL-101 particles. The mechanism of hydroxyl radical reaction with terephthalic acid (PTA) was used to determine the content of CuO_x_@MIL-101 hydroxyl radical (typically reactive oxygen species) production. The peak intensities at 426 nm in photoluminescence spectra are respect to the hydroxyl radical levels in solution. As shown in Figure 1D, with the increase of reaction time, the fluorescence intensity gradually increased, proving that the production of hydroxyl radicals increased. In general, the newly designed MOFs (CuOx@MIL-101), which are synthesized using a chromium cluster and terphenyl acid as photosensitizer components, possess several advantages. These include exceptional stability, ample space, and a high yield of hydroxyl radicals. When exposed to visible light, a large number of hydroxyl radicals are generated, which subsequently leads to cell death and decreased collagen deposition. In contrast, traditional PDT for scar treatment using 5-ALA requires specific wavelength light conditions and has a relatively low rate of ROS generation (Tosa and Ogawa, 2020).

### 3.2 *In vitro* CQ-assisted PDT of HSFs

#### 3.2.1 CuO_x_@MIL-101 was biocompatible *in vitro*

**FIGURE 2:**
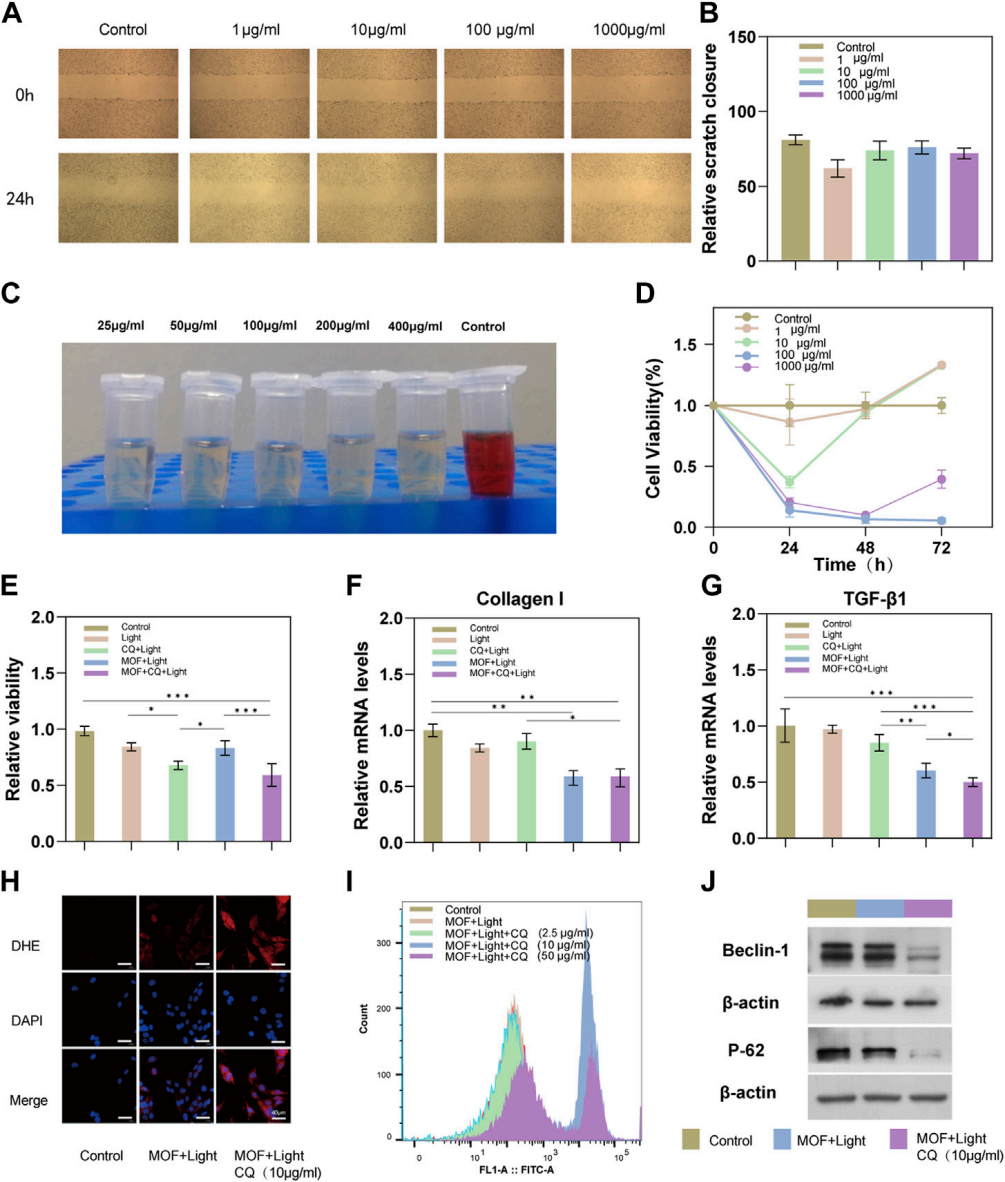
Biocompatibility and PDT therapeutic efficacy of MOFs (CuO_x_@MIL-101). **(A)** Scratch-wound assay to evaluate the migration of HSFs treated with different concentrations of MOFs. **(B)** Quantification of scratch wound closure after 24 h treatment. **(C)** Hemolysis assays were conducted to evaluate the safety of MOFs in different concentrations (25, 50, 100, 200, and 400 μg mL^−1^). **(D)** Viability of HSFs after treatment with different concentrations of MOFs for 24, 48, and 72 h. **(E)** Viability of HSFs after treatment with Light, CQ + MOFs, MOFs + Light, and MOFs + Light + CQ for 24 h. **(F, G)** Relative mRNA expressions of TGF-β1 and collagen I in HSFs treated by Light, CQ + MOF, MOFs + Light, and MOFs + Light + CQ for 24 h. **(H)** Intracellular ROS was measured using ROS Fluorescent Probe-dihydroethidium (DHE) (scale bar: 40 μm) **(I)** ROS production was measured with the flow cytometric analysis. (n = 3, * *P* <0.05; ** *P* <0.01; *** *P* <0.001). **(J)** Western blotting assay was carried out to detect expression levels of autophagy-related proteins (Beclin 1, and P62).

In this section, the biocompatibility of CuO_x_@MIL-101 was verified using different dimensions. As shown in Figure 2A, HSFs was treated with different concentrations of CuOx@MIL-101 to evaluate its migration ability. Due to the rapid migration of HSFs in the control group (0 g/ml CuO_x_@MIL-101), the scratch healed rapidly in the control group (Figure 2A, B). By comparison, HSFs migration was obviously inhibited in the 10 μg/ml CuO_x_@MIL-101 group. As shown in Figure 2D, the cytotoxicity was found to be dependent on concentration, with the optimal range being 1–10 μg/ml. Therefore, 3 μg/ml of CuOx@MIL-101 was selected for further experimentation. Different concentrations of CuO_x_@MIL-101 were then incubated with red blood cells at 37°C for 8 h. In Figure 2C, the supernatant of the experimental group was transparent in comparison with that of the positive control group, indicating that CuO_x_@MIL-101 had almost no hemolysis occurred. Taken together, the CuO_x_@MIL-101 used in this study had good biocompatibility.

#### 3.2.2 The suppressive effect on cell proliferation of HSFs

In the initial stage, we identified the optimal concentration of CQ for the experiment to be between 2.5 and 10 μg/ml (Supplementary Figure S2). To achieve maximum efficiency in inhibiting autophagy, we selected 10 μg/ml as the concentration of CQ for subsequent experiments. Additionally, the optimal duration of light therapy was determined to be 5 min (Supplementary Figure S3). Additionally, we compared the therapeutic effects of two other autophagy inhibitors (HCQ and 3-MA) on HSFs. The HSFs proliferation inhibition efficacy was more stable with increasing concentrations of CQ (Supplementary Figure S4). Typically, CQ is able to inhibit autophagy by binding to lysosomes and neutralizing their acidity. This prevents the fusion of autophagosomes with lysosomes and the subsequent degradation of autophagic cargo, leading to the accumulation of autophagosomes and a decrease in autophagic flux (Yao et al., 2022). According to Figure 2E, combined with the control group, the PDT combined with CQ treatment group had the lowest cell survival rate (63% of the control group), while the PDT group had a relatively higher cell survival rate, probably because PDT could initiate protective autophagy of cells. A statistical analysis revealed a significant difference between the experimental group treated with PDT + CQ and the PDT group and the CQ group (*p* < 0.05), indicating that the addition of the autophagy inhibitor CQ in the photodynamic therapy became the sharp edge of PDT, thereby improving its efficacy. Furthermore, the inhibition results of the CQ + L, MOF + L, and MOF + CQ + L groups on cell proliferation align with our expectations. This suggests that CQ effectively inhibits autophagy. We can therefore conclude that the drug toxicity of CQ did not impact the experimental results.

#### 3.2.3 Expression of genes responsible for collagen proliferation and inflammatory response in HSFs

Inhibition of inflammation and collagen hyperplasia in scar tissue is an important means of treating scars (Berman et al., 2017). A test of the expression of genes related to TGF-1 and collagen I was carried out in scar fibroblasts treated according to the group’s guidelines. As shown in Figure 2F–G, the RNA expression of Collagen I and TGF-β in scar fibroblasts of the PDT combined with the chloroquine group and the PDT group was significantly downregulated, indicating that the two treatment methods inhibited the production of type I collagen, especially in the combined treatment group.

#### 3.2.4 Expression of proteins involved in collagen synthesis and autophagy in HSFs

HS is a fibroproliferative disease caused by the excessive proliferation of fibroblasts and ECM during wound healing (Zhang et al., 2020). Previous studies have demonstrated that transforming growth factor-β1 (TGF-β1) has a fibrogenic effect in hypertrophic scar fibrosis and plays a crucial part in fibroblast differentiation by regulating the expression of α-smooth muscle actin (α-SMA) in fibroblasts (Lu et al., 2005; Qian et al., 2022). α-SMA is a typical marker of the fibroblast contraction phenotype. Myofibroblasts rich in α-SMA are an important pathological basis for HS (Arora et al., 1999). Activated by TGF-β1, α-SMA-positive myofibroblasts can synthesize and secrete ECM, which further aggravates HS fibrosis (Aarabi et al., 2007). In scar tissue, AMPK has been found to be activated and it can upregulate collagen production in fibroblasts. Activation of AMPK also leads to the phosphorylation and activation of downstream targets such as p38 and Akt (Zhang et al., 2020). These downstream targets have been found to promote fibroblast activation and differentiation into myofibroblasts which are responsible for the excessive collagen production and extracellular matrix deposition in scars (Zhang et al., 2020). As expected, the expression of α-SMA was evaluated when the HSFs were administered with MOF + light irradiation, and it was found that this expression can be reversed by the presence of CQ (Supplementary Figure S5). These results strongly suggest that CQ-assist PDT can effectively decrease collagen synthesis in hypertrophic scars (HS).

In recent years, PDT has become an effective means of treating hypertrophic scars (Zhang et al., 2019; Chen Y. et al., 2021). However, studies have shown that PDT induces autophagy in a dose-dependent manner and insufficient ROS production during PDT can induce protective autophagy in HSFs (Huang et al., 2022b). LC3II/I, Beclin 1 and P62 are classical autophagy-related proteins. The maturation of autophagosomes is marked by the conversion of LC3-I to LC3-II (Pozuelo-Rubio, 2011a; Pozuelo-Rubio, 2011b; Cai et al., 2018). Therefore, LC3-II/I ratio can be used to assess autophagy. As a specific substrate of autophagy, p62 is degraded during autophagy; therefore, the intracellular level of p62 acts as a marker of autophagic flux (Klionsky et al., 2008; Rezabakhsh et al., 2018). Beclin 1 is a protein that plays a vital role in the initiation of autophagosome formation. It functions as a scaffold protein, assembling the necessary proteins for autophagosome formation and is essential for the formation of the initial pre-autophagosomal structure. The activity of Beclin 1 is regulated by various post-translational modifications and is crucial for the formation of autophagosomes. Therefore, the level of Beclin 1 protein or its activity can be used as a marker to indicate the level of autophagy in the cell (Yue et al., 2003; Fu et al., 2013).

Chloroquine (CQ), a classic autophagy inhibitor, was introduced into the treatment group to interfere with the protective autophagy of cells by preventing autophagosomes from fusing with lysosomes, resulting in the accumulation of mature, ineffective autophagic vacuoles (Redmann et al., 2017; Mauthe et al., 2018). In each group of cells, Beclin 1, LC3II/I and P62 expression was detected by western blotting (Figure 2J). An increase in the conversion of Beclin 1 was observed in the group treated with MOFs + Light + CQ, along with a increase in P62 levels. However, this increase in Beclin 1 conversion and P62 levels was reversed by the addition of CQ treatment. Despite this reversal, the level of LC3II/I was increased in the MOFs + Light + CQ group (Supplementary Figure S5). This may be due to the fact that CQ increases the acidification of lysosomes, leading to an accumulation of LC3II/LC3I in these organelles (Manic et al., 2014). This confirms that PDTcan cause cell autophagy in HSFs and that CQ can effectively block autophagosomes from fusing with lysosomes. Together, these results demonstrate that PDT induces protective autophagy in cells, thereby enhancing its anti-scarring effect.

#### 3.2.5 Analysis of ROS generation in HSFs

Previous research has demonstrated that Photodynamic Therapy (PDT) can effectively increase the production of ROS in cells, leading to apoptosis (Miao et al., 2021; Zhao et al., 2021). As shown in Supplementary Figure S6, apoptosis was reduced by 5-ALA mediated PDT for HSFs when its concentration was below 4 mM. According to the mechanism of PDT, apoptosis is mostly caused by the generation of cytotoxic ROS (Li et al., 2006), and an autophagy inhibitor can block the protective autophagy induced by PDT and reduce ROS consumption (Han et al., 2017; Mohammadi et al., 2017). Therefore, ROS production of CuO_x_@MIL-101 was evaluated by flow cytometry. As expected, under light irradiation, showed that ROS production was enhanced when CuO_x_@MIL-101 + CQ was co-cultured with HSFs under light irradiation (Figure 2I). The separate images and quantitative analysis were in the Supplementary Figure S7. The Similar results were illustrated in the view of DHE probe (Figure 2H). Overall, the results argue that CuO_x_@MIL-101 catalyzed the production of intracellular ROS following visible-light irradiation. Furthermore, the addition of CQ would boost ROS generation by inhibiting pro-survival autophagy of PDT, and resulting in optimized anti-scarring effect.

### 3.3 MNP morphology and characterization

**FIGURE 3:**
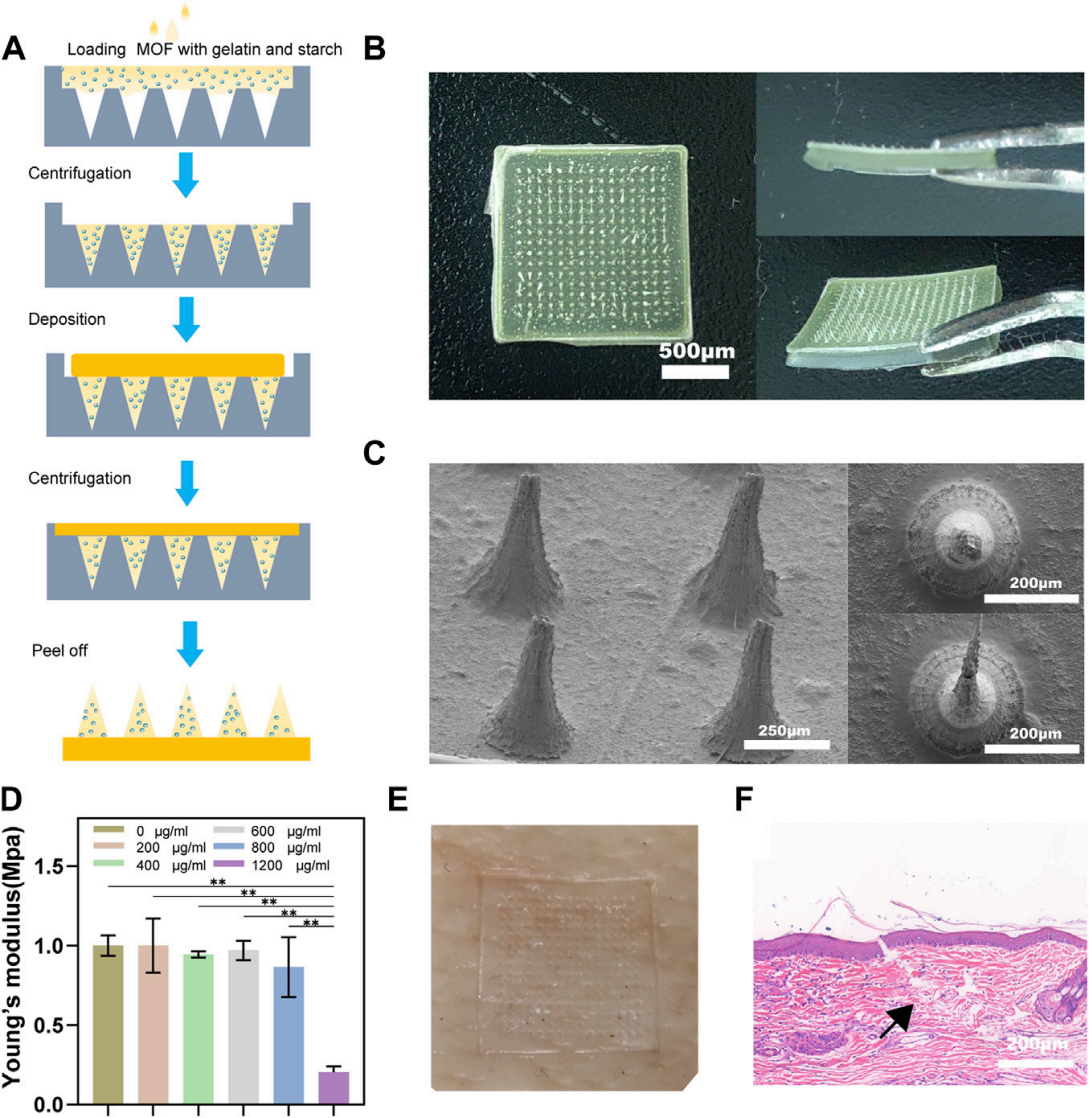
Morphology and mechanical properties of microneedle patches. **(A)** The schematic fabrication process of microneedle patches. **(B)** Optical images of microneedle patches, including a vertical view of a patch, and a cross-sectional view of a patch. **(C)** SEM images of microneedle patches with different magnifications. **(D)** Young's modulus of MNs with different MOF concentrations. **(E)** After pressing the pig skin with the needle array, blue dye was rubbed on the skin. **(F)** H&E staining image of rabbit HS after insertion with MN patch. The black arrow indicates the broken stratum corneum of the skin.

For HS to be effectively treated, drugs must penetrate the dermal layer of the skin. Compared with normal skin, the cuticle, epidermis, and dermis of HS are all thickened to varying degrees, and excessive deposition of ECM makes the tissue denser, which greatly hinders the transdermal absorption of drugs (Prausnitz and Langer, 2008; Han et al., 2020). Accordingly, MNP with excellent mechanical strength and permeability are ideal dressings for transdermal drug delivery (Indermun et al., 2014; Wen et al., 2021). Our MNP made of gelatin and starch has good mechanical properties and is sufficient to penetrate scar tissue and reach the dermis of the skin. A schematic of the fabrication process of a complete MNP is shown in Figure 3A, and we can see that the loaded MOFs and CQ were mainly deposited in the needle body. Figure 3B shows a general view of the MNP. Photoresist technology was used to prepare the microneedle array mold (needle height 800µm, base diameter 240 µm, tip diameter 10 μm, center spacing 700 μm, and array number of microneedles 15 × 15. The morphology of the MNP under the scanning electron microscope is shown in Figure 3C, where a series of microneedles were evenly distributed on the base to form a microneedle array. MNP has the ability to penetrate the skin and tissue barriers. In order to determine the mechanical strength of MNP, different concentrations of CuOx@MIL-101 were designed and manufactured, resulting in significant differences in the morphology and characteristics of the MNP (Supplementary Figure S8). CuO_x_@MIL-101 with a concentration of 1000 μg/ml was not suitable for fabricating MNP from optical and gross images because of their fragile needle bodies. Meanwhile, with CuO_x_@MIL-101 concentration over 1000 μg/ml, Young’s modulus of the hydrogel MNP was remarkably increased to nearly 1 MPa, illustrating the poor mechanical strength of the MNP (Figure 3D).

To further verify the mechanical strength of the MNP, pig skin was used to punctured. After the acupuncture and removed the MNP, visible channels appeared on the pig skin (Figure 3E). We established a rabbit ear scar model and punctures were performed using the MNP described above. Figure 3F shows the HE staining results, indicating that the depth of the microneedle piercing the rabbit ear scar was approximately 300 μm, which also confirmed that the microneedle had penetrated the cuticle of the scar. All these results demonstrated that the MNP have sufficient mechanical strength to penetrate skin barrier for delivering photosensitizers into the scar.

### 3.4 *In vivo* study of PDT treatment with MNP combined with CQ

#### 3.4.1 Rabbit ear model of scar

**FIGURE 4:**
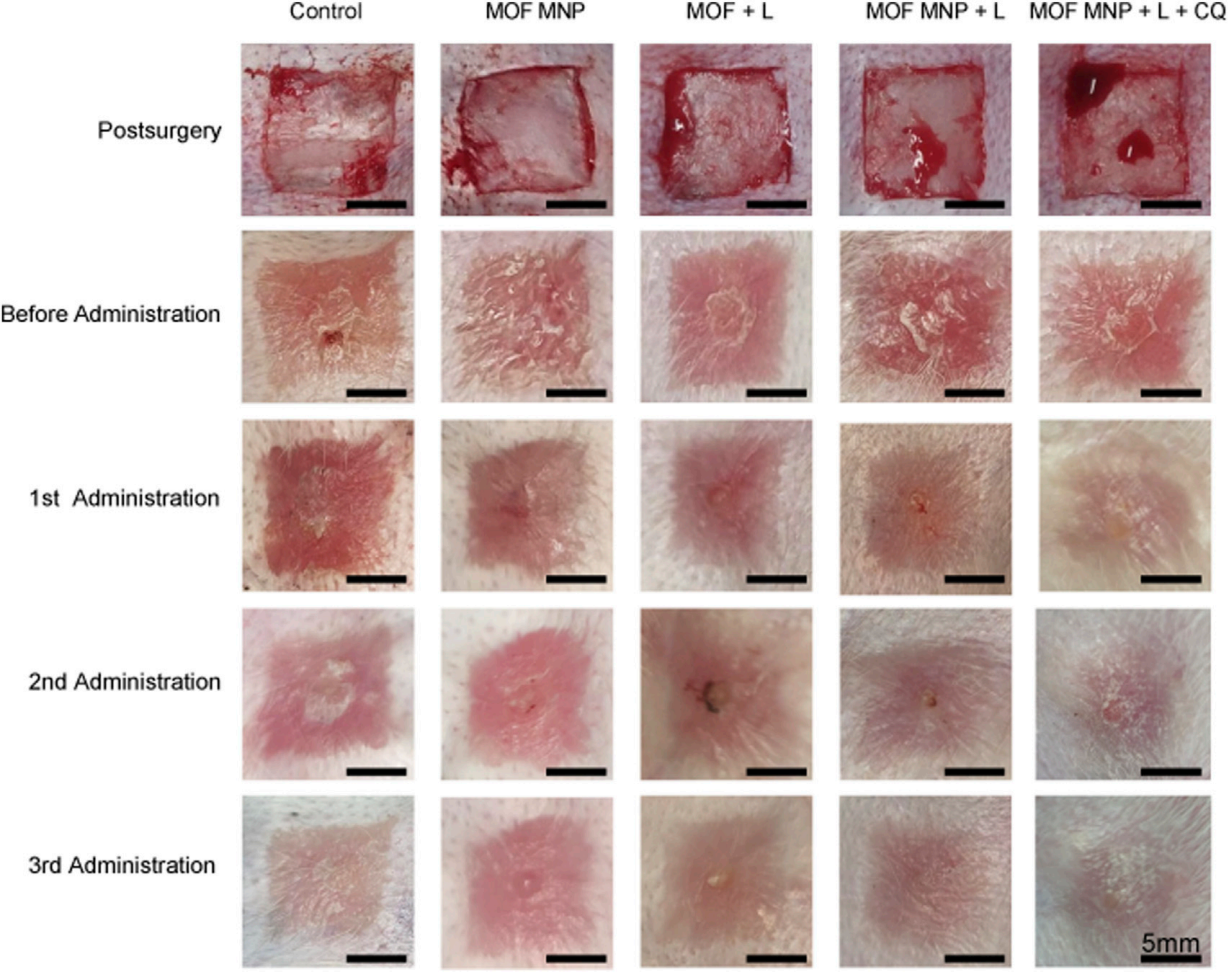
Treatment effect of rabbit HSs by different groups. (L: light, *n* = 4).

To investigate the efficacy of MNP in scar PDT treatment, we established a rabbit ear scar model. Figure 4 shows the gross images immediately after modeling. After 21 days post-surgery (the total time to establish the HS model), the treatment effect of CuO_x_@MIL-101/CQ-loaded MNP on rabbits HSs was investigated using five treatment groups: MOFs MNP, MOFs + light, MOFs MNP + light, MOFs MNP + CQ + light, and a blank treatment group. After three administrations, the ear scars in the control group were raised and darker in color, while the rabbit ear scar in the MOFs MNP + CQ + light group was significantly flatter than that in the other treatment groups. MOFs MNP and MOFs + light did not significantly differ from the control group in their effects, indicating that simple MOFs smearing on rabbit ear scars for PDT and MOFs MNP treatment cannot eliminate the scars. The scar in the MOFs MNP + light group was slightly improved after treatment, but it remained dark red and had little effect. Among all the treatment groups, the rabbit ear scar after treatment in the MOFs MNP + CQ + light group was closest to the normal skin.

#### 3.4.2 Histological analysis

HS is a skin fibroproliferative disease characterized by excessive proliferation of dermal fibroblasts, excessive deposition of dermal collagen fibers, extracellular matrix protein deposition, and epithelial-mesenchymal transition (EMT) (Zhang et al., 2016; Lee and Jang, 2018). We evaluated the status of HS using microscopic observation of histopathological features in the different treatment groups. The scars were stained with HE and Masson’s trichrome to observe histological changes and quantitatively evaluate SEI and CVF.

**FIGURE 5:**
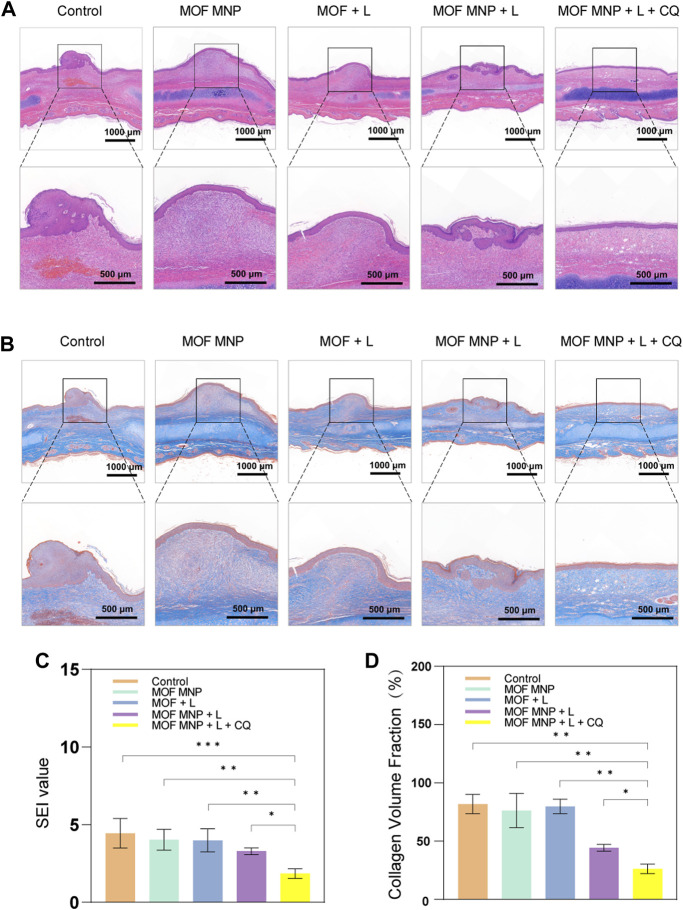
Histological analysis of HSs with different treatments. **(A)** Representative H&E staining images of rabbit HSs after different treatments. **(B)** Masson's staining images of rabbit HSs after different treatments. **(C)** SEI and **(D)** CVF of HSs after different treatments. (*n* = 4, *P <0.05; **P <0.01; ***P <0.001).

Figure 5A shows the results of HE staining. The number of fibroblasts in the MOFs MNP + CQ + light group is the least, and the other groups have different degrees of neutrophil infiltration. The SEI results for each group are shown in Figure 5C. As predicted, control group SEI was significantly higher, and MOFs MNP and MOFs + light results were not significantly different from control group SEI. Consistent with the results presented in the general photographs of rabbit ear scars after treatment, the SEI of the MOFs MNP + light group was lower than that of the PDT group treated with MOFs alone, but the difference was not significant. However, the SEI of the MOFs MNP + CQ + light group was significantly reduced, indicating that the MOFs was introduced into the deep scar tissue by microneedles, and the MOFs (CuO_x_@MIL-101) based PDT indicated a good an inhibitory effect on the formation and growth of hypertrophic scars.

Additionally, Masson staining showed (Figure 5B) that compared with the dense, curly, and irregular collagen arrangement in the blank control group, the collagen arrangement in the scar tissue of the MOFs MNP + CQ + light group was looser and more regular, and the direction was consistent with the epidermis. Figure 5D shows the quantitative measurement of collagen volume, and the results also show that MOFs MNP combined with PDT treatment can significantly improve collagen deposition in scar tissue. Notably, the best inhibitory effect was realized in the combined treatment by MOF-assisted PDT and CQ. As a result, the collagen deposition process was blocked by the MOF-assisted PDT and its anti-scarring efficacy can be boosted by CQ.

## 4 Conclusion

Our study presents a thoroughly-constructed functionalized microneedle delivery system loaded with the MOFs and autophagy inhibitor as a viable and efficient protocol for the treatment of hypertrophic scars using daylight photodynamic therapy (dPDT). The use of gelatin and starch as substrates for the MNP imparts high mechanical strength, enabling them to puncture the corneum and extracellular matrix on the scar surface. The robustness and transdermal efficacy of the microneedles were successfully demonstrated. Furthermore, the MOFs exhibited exceptional physical and chemical properties, capable of generating high levels of ROS under visible light and thereby enhancing the efficacy of PDT. Notably, PDT-induced ROS eliminated hypertrophic scar fibroblasts while also triggering protective autophagy in the cells. The inclusion of the autophagy inhibitor CQ in the microneedles effectively suppressed this process, resulting in the death of HS fibroblasts and the reduction of collagen deposition.

In summary, this study represents a pioneering effort in the practical application of MOFs, which possess photocatalytic properties. By fully leveraging the designable structural characteristics of MOFs, the research combines them with scar PDT under visible light conditions. By considering the impact of cellular autophagy on the effectiveness of photodynamic therapy, the use of microneedles as a delivery vehicle to combine autophagy inhibitors and photosensitizers enhances the therapeutic outcomes of PDT. This functionalized MNP advocates a new combined application of PDT with visible-light and provides a novel strategy for dPDT in hypertrophic scar treatment, which has a very high potential for clinical application.

## Data Availability

The original contributions presented in the study are included in the article/Supplementary Material, further inquiries can be directed to the corresponding authors.
