# Supplementary material for: Visible light-driven photodynamic therapy for hypertrophic scars with MOF armored microneedles patch
Source: Front Chem. 2023 Feb 16;11:1128255. doi: 10.3389/fchem.2023.1128255 (PMC9978826; doi:10.3389/fchem.2023.1128255)
Supplement: Supplementary file 1 [file DataSheet1.docx]

**Visible Light-Driven Photodynamic Therapy for Hypertrophic Scars with MOF Armored Microneedles Patch**

**Danyang Chen^1†^, Yixuan Zhang^1†^, Wei Long^1^, Langjie Chai^1^, Thazin Phoone Myint^1^, Wei Zhou^1^, Ling Zhou^1^, Min Wang^1*^, Liang Guo^1*^**

^1^ Department of Plastic Surgery, Zhongnan Hospital of Wuhan University, Wuhan 430071, China

**^†^**These authors contributed equally to this work.

*** Correspondence:**Liang Guo
guolianghbwh@163.com

Min Wang

sakurawangmin@whu.edu.cn

**Supplementary Figures and Tables**

**
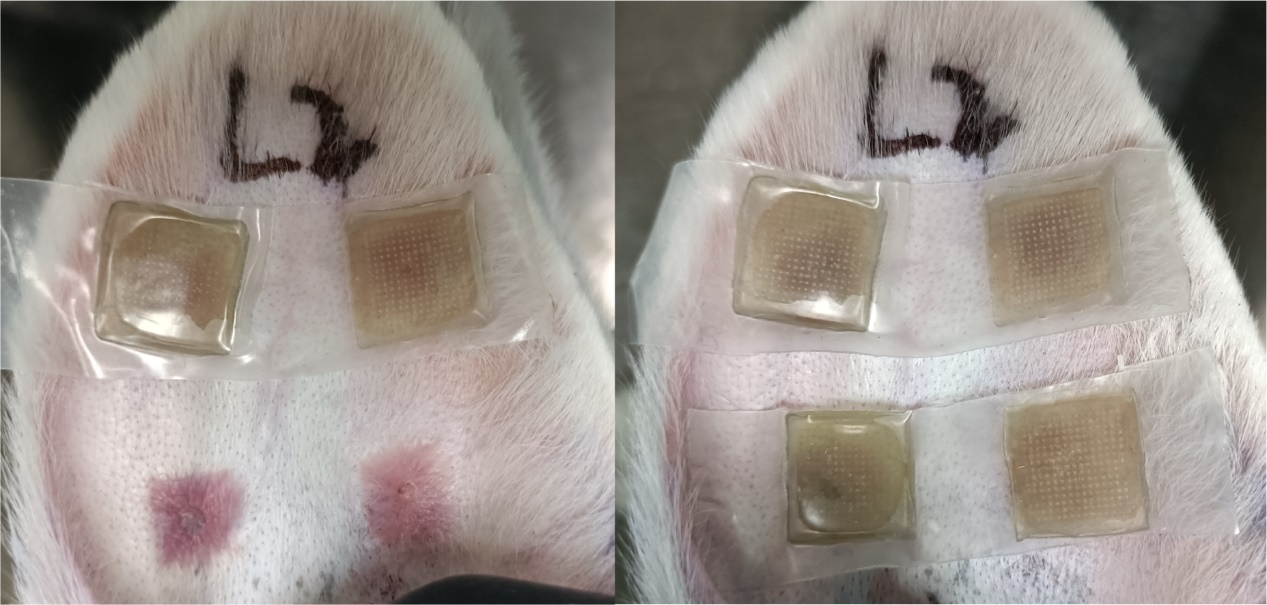
**

**Figure S1.** The therapeutic effect of MNP on rabbit ear scar (groups: MOFs MNP, MOFs + light, MOFs MNP + light, and MOFs MNP + CQ + light)


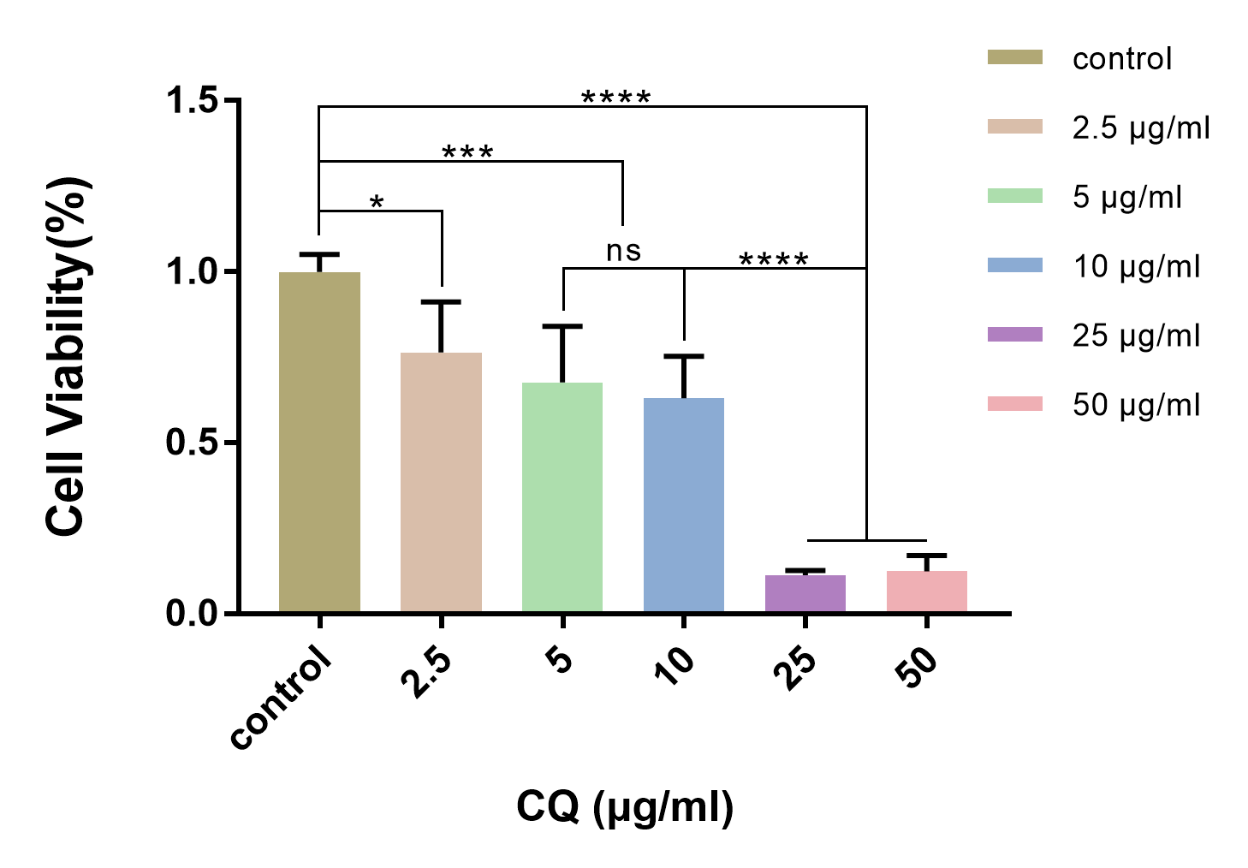


**Figure S2.** Screening of concentrations used for autophagy inhibitor chloroquine. (n=3, ^*^ P < 0.05; ^**^ P < 0.01; ^***^ P < 0.001).


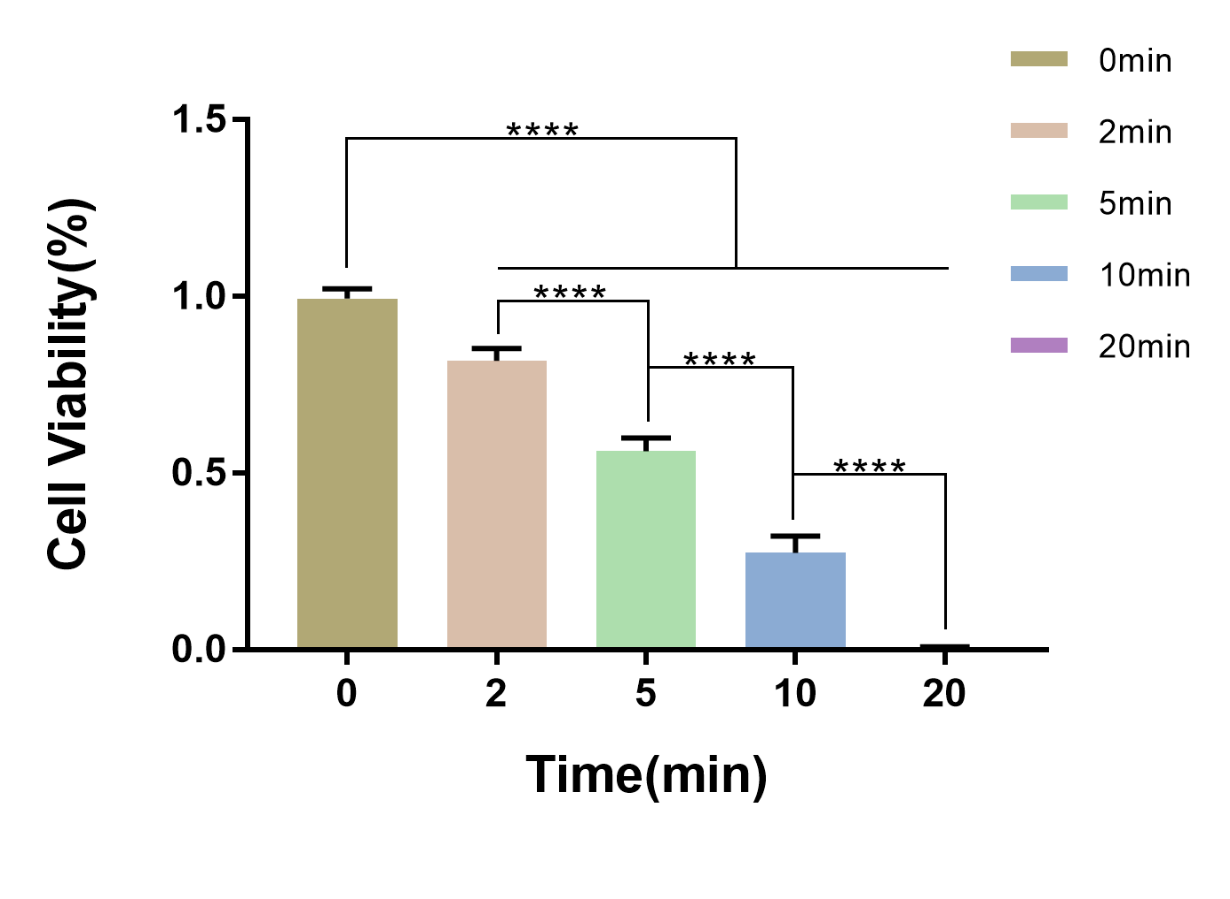


**Figure S3.** Toxicity of CuO_x_@MIL-101 to hypertrophic scar fibroblasts (HSFs) under different light times. (n=3, ^*^ P < 0.05; ^**^ P < 0.01; ^***^ P < 0.001).


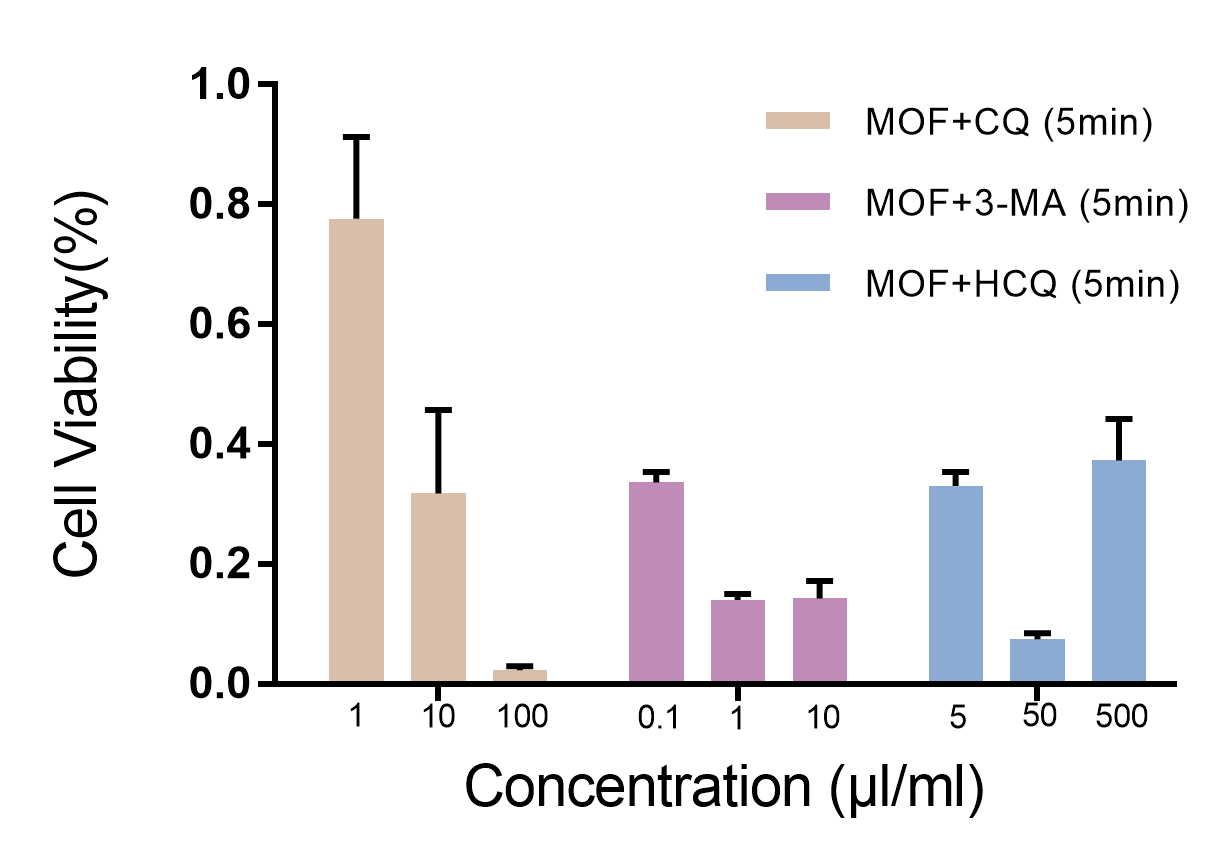


**Figure S4.** HSFs proliferation inhibition of PDT (light duration 5min) with three autophagy inhibitors combined with CuO_x_@MIL-101 on HSFs. (n=3, ^*^ P < 0.05; ^**^ P < 0.01; ^***^ P < 0.001, 3-MA:3-Methyladenine; HCQ: Hydroxychloroquine).


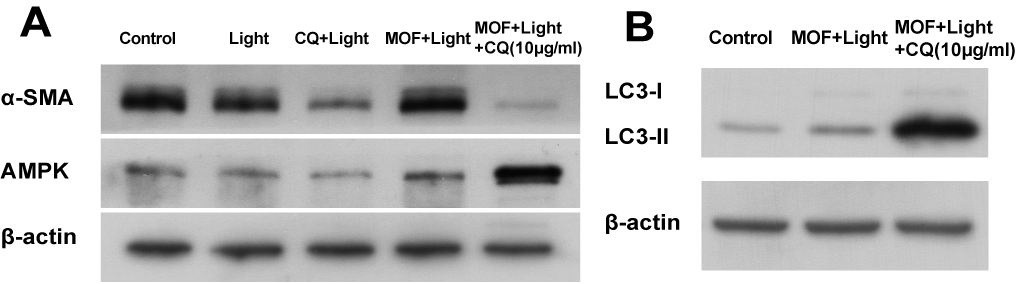


**Figure S5** Western blotting assay was carried out to detect expression levels of α-SMA, AMPK, and LC3II/I.


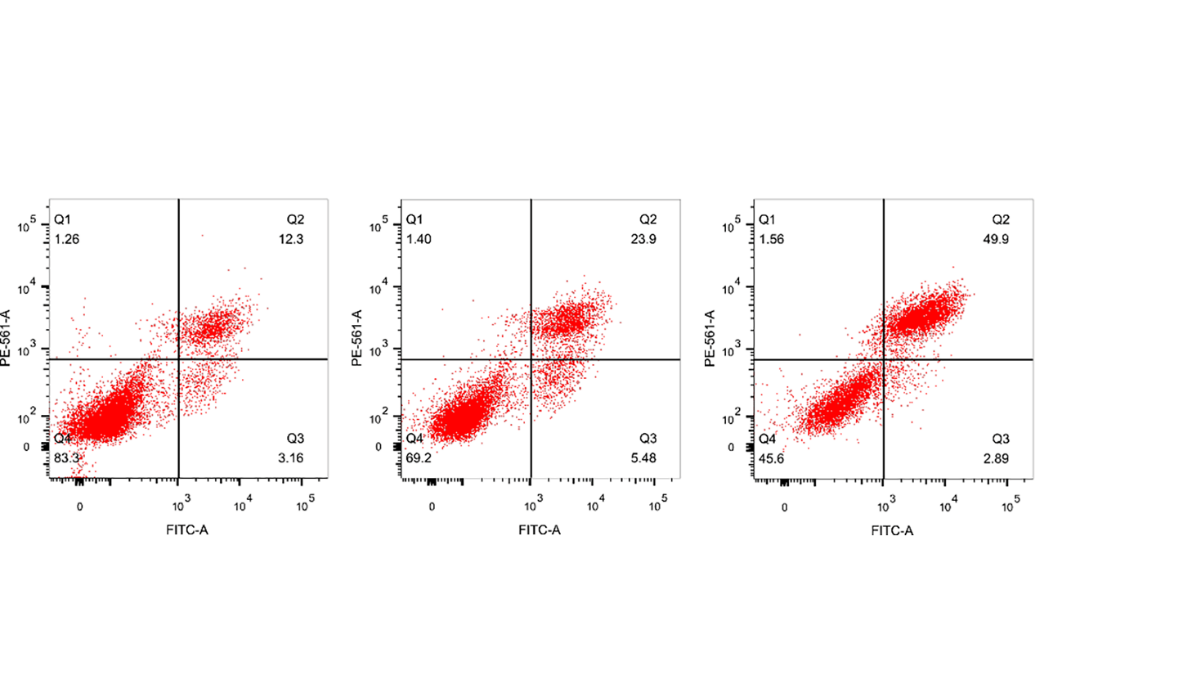


**Figure S6.** The PDT increased the apoptosis of HSFs. Annexin V-FITC was used to detect the apoptosis level of different treatment groups (Control, CuO_x_@MIL-101+light, CuO_x_@MIL-101+ CQ+Light)


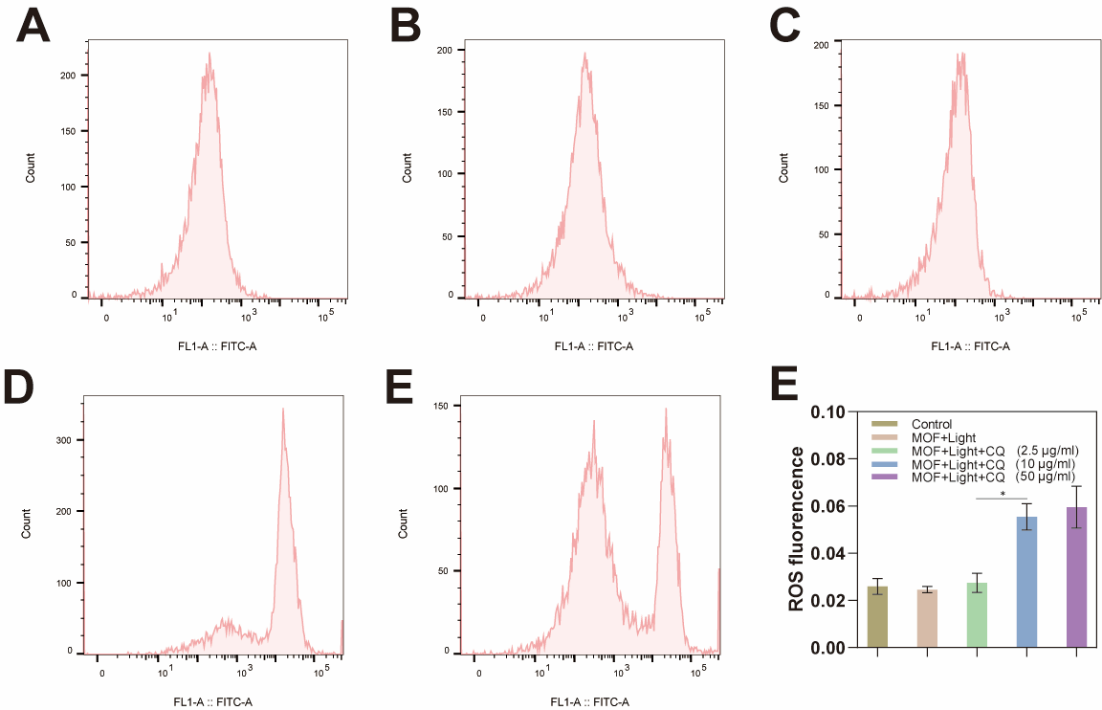


**Figure S7** ROS generation of CuOx@MIL-101 was evaluated by flow cytometry. HSFs were treated treat with CuOx@MIL-101+light irradiation with different concentrations of CQ. The flow cytometry data demonstrated that the ROS generation was distinctly enhanced with the increase of CQ concentration under 10 μg/ml.


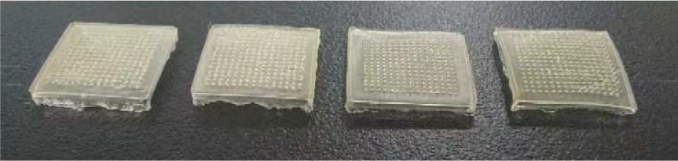


**Figure S8.** Optical images of microneedle patches prepared with different MOF concentrations, including materials of 200μg/ml, 400μg/ml, 200μg/ml, and 1000μg/ml, respectively.

**
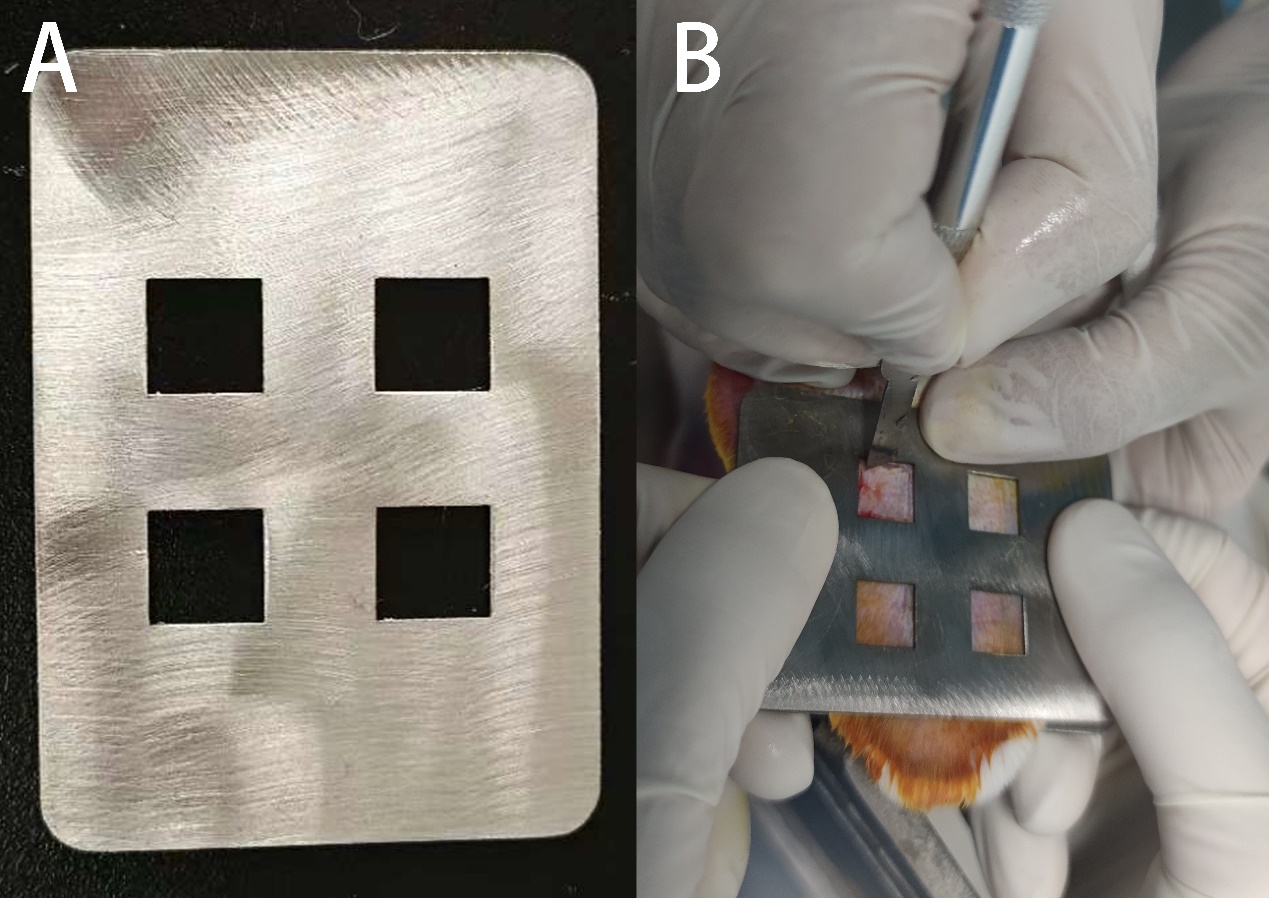
**

**Figure S9.** A stainless steel plate with 4 square holes was applied as a mold to make sure a 1 × 1 cm square area for each rabbit ear scar.

**Experimental Procedures**

**Hypertrophic scar fibroblast harvest and cell cultivation**: In brief, tissue specimens were washed sequentially with phosphate-buffer saline (PBS) and 1% (v/v) penicillin–streptomycin to remove blood stains. After that, it was treated with neutral protease to remove epidermis. The tissue was then cut into pieces, soaked in collagenase I, and filtered. The supernatant was removed and resuspended by centrifugation, which was repeated twice, and the cell precipitate was resuspended in Dulbecco's Modified Eagle medium (Invitgen) supplemented with 10% (v/v) FBS and 1% (v/v) penicillin–streptomycin 37 °C in an incubator with 5% CO2 and 95% relative humidity. The culture medium was changed every 48 hours and passed every 4–5 days to maintain a logarithmic growth.

**Hemolysis assay:** different concentrations of CuOx@MIL-101 (25, 50, 100, 200, and 400 μg mL^−1^) in 1 mL PBS solution were mixed with 200 μL of 10% erythrocytes (v/v) and incubated for 8 h at 37 °C. The double-distilled water mixed with erythrocytes was set as the control group. The presence or absence of hemolysis in each group of tubes was observed by visual inspection of erythrocyte fragmentation and sedimentation.

**PCR assay**： Reverse transcription was performed using the HiScrip III RT SuperMix reverse transcription kit and quantitative polymerase chain reaction (qPCR) was performed according to the manufacturer's protocol. After reverse transcription, Real-Time PCR was performed using a 7500 real-time PCR system with ChamQ SYBR qPCR Master Mix. The expression of each gene was normalized to that of GAPDH. For amplification, the following primer sequences were used: GAPDH (Forward, TGA AGG TCG GAG TCA ACG GAT TTG; Reverse, CAT GTG GGC CAT GAG GTC CAC CAC ); TGF-β1 (Forward, GGA AAT TGA GGG CTT TCG CC; Reverse, CCG GTA GTG AAC CCG TTG AT); Collagen I (Forward, GAG GGC CAA GAC GAA GAC ATC; Reverse, CAG ATC ACG TCA TCG CAC AAC); IL-6 (Forward, ACT CAC CTC TTC AGA ACG AAT TG; Reverse, CCA TCT TTG GAA GGT TCA GGT TG); Smad3 (Forward, GCG CAC TGA CCA TAA GAG CA; Reverse, ATC CAG GGA CTC AAA CGT GG)

**Histological analysis and immunostaining:** ear scar specimens were collected and fixed with 4% (v/v) paraformaldehyde. Dehydrated using a graded series of ethanol solutions (70%, 85%, 90%, 95%, and 100%) and xylene (50% and 100%), embedded in paraffin, and sectioned using a Leica RM2245 microslicer (Leica Microsystems, Wezlar, Germany). All specimens were stained with H&E, Masson's trichrome (Sigma-Aldrich Corp., St.Louis, MO, USA). The epithelialized collagen regeneration of the sample tissue was observed under inverted and upright fluorescence microscopy (Olympus BX51) .

**SEI calculation:** The scar hyperplasia index (SEI) value was calculated according to HE staining images to assess HSs as follows: SEI = H/H0, where H was the height from the highest point of the HSs to the cartilage surface, and H0 was the height from the stratum corneum to the cartilage surface of adjacent normal skin. The SEI was taken as the mean of 5 HSs in each group. Scarring was assessed as an SEI value > 1.5. Collagen deposition levels in HSs were assessed by collagen volume fraction (CVF). CVF (the ratio of collagen area to total area in Masson stained images) was calculated by Image J software, and 5 HSs were used for CVF analysis in each treatment group.

**Measurement of CuOx@MIL-101 hydroxyl radical production:** Briefly, 10 mg samples were well dispersed in 50 mL terephthalic acid (PTA) aqueous solution (containing 0.5 mM PTA and 1.5 mM NaOH, pH = 7) in the dark at room temperature. Then a 300 W Xe lamp (Beijing PerfectLight) with light filter (λ > 420 nm) is employed at the top of the solution as a light source at a distance of 10.0 cm. During irradiation, the photoluminescence (PL) spectra of the solution is examined every 15 minutes by the fluorescence spectrophotometer (Hitachi Model F-4600 FL Spectrophotometer) under excitation with UV light at 315 nm. And the peak intensities at 426nm in photoluminescence spectra are respect to the hydroxyl radical levels in the solution.
